# Identification and characterization of microRNAs in the liver of yak (*Bos grunniens*) associated with energy deficiency at high-altitude

**DOI:** 10.5713/ab.24.0756

**Published:** 2025-04-01

**Authors:** Shuru Cheng, Fangping Shi, Zhixiong Tang, Feifei Yang, Quanlu Meng, Ting Liu

**Affiliations:** 1 College of Animal Science and Technology, Gansu Agricultural University, Lanzhou, China; 2 Helan County People’s Hospital, Yinchuan, China; 3 College of Biological and Architectural Engineering, Baoji Vocational & Technical College, Baoji, China

**Keywords:** High Altitude, Liver, MiRNA, Winter Grazing, Yak

## Abstract

**Objective:**

MicroRNAs (miRNAs) are small non-coding RNAs that regulate various biological processes including systemic metabolism and energy homeostasis. Comprehending hepatic functional adaptations is critical for yak (*Bos grunniens*) cattle as these herds typically suffer from malnutrition during the most of winter grazing seasons.

**Methods:**

To enhance the understanding of miRNA mediated post-transcriptional regulation in the context of energy deficiency in yaks, the miRNA transcriptomes of liver were profiled using deep sequencing analyses.

**Results:**

A total of 17,562,643 clean reads were acquired from a liver library, resulting in the identification of 369 known and 41 novel miRNA candidates. The top 10 miRNAs accounted for greater than 92% (9,854,350 counts) of the miRNAome, which were predicted to target 2,333 genes. Among the highly expressed miRNAs, miR-122 accounts for 57%, followed by bta-let-7f and bta-let-7a-5p. Gene Ontology and Kyoto Encyclopedia of Genes and Genomes pathway analyses using DAVID bioinformatics resources indicated that the identified miRNAs targeted genes enriched in transcription and metabolism processes. Notably, 11 of the top 20 miRNAs were predicted to play a role in the regulation of lipid as well as glucose homeostasis via the PI3K-Akt interaction network.

**Conclusion:**

This is the first, at the best of our knowledge, liver miRNA-seq profiling analysis of moderate yak cows subjected to the combined effects of cold and nutritional stress at high altitude. Tissue specific miR-122 and liver enriched bta-let-7f represent two of the most abundant miRNAs found in the liver of yak cows. These findings enhance our understanding of the role of hepatic miRNAs in energy deficiency in yaks during winter grazing at high altitudes.

## INTRODUCTION

Vertebrate animals display remarkable ability to tolerate high altitudes and compensate for the combined effects of cold and concomitant decreases in O_2_ supply that potentially constrain aerobic metabolism [1]. The yak (*Bos grunniens*) is regarded as a typical seasonal grazing ruminant inhabiting in remote mountain regions of altitudes ranging from 3,000 to 5,500 m throughout the Hindu Kush-Himalayan and the Qinghai-Tibetan Plateau (QTP). More than 14 million domestic yaks provide the local Tibetan pastoralists with necessary resources and financial income, such as meat, milk, transportation, dung for fuel and hides for tented accommodation [2]. The alpine highlands where the yak is found characterised by severe climate at high altitudes and with grazing resources restricted by very short growing seasons. As the forage supply decreases sharply in winter, the herds generally suffer from malnutrition for almost 8 months of the year, and the resulting energy deficiency.

In mammals, the liver integrates nutrients uptake and circulating carbohydrates and lipids to peripheral tissues for the function of maintaining overall organism energy balance [3]. Low production, reduced fertility and immune related are known to be effected by energy deficiency in yaks [4]. During and prolonged starvation in the whole winter grazing seasons, an enhanced rate of gluconeogenesis that utilizes nonglycosidic intermediates such as lactate and pyruvate to produce glucose [5], in addition an increased hepatic fatty acid oxidation [6] are needed to provide the yak with insulation from cold as well as an energy reserve. Our previous study explored the effects of metabolites and environmental factors on lipid deposition and metabolism in liver and adipose tissues of yaks, provided the first example that the regulation of lipoprotein lipase involves some factors in addition to insulin and triglycerides for yak to better adapt to the harsh environment [7]. With the liver being the central metabolic organ in the body and is therefore assumed to be relatively vulnerable to hypoxemia [8]. Little is known, however, about the combined effects of cold exposure and nutrients stress on hepatic functional adaptations in this animal that native to high altitude. We hypothesized that hepatocytes maintain metabolic homeostasis by coordinating gene expression programs in response to dietary and systemic signals for the yaks.

It is well known that microRNAs (miRNAs) are a class of endogenous non-coding small RNAs of ~22 nucleotides in length found in most eukaryotes, which function as transcriptional and post-transcriptional regulators of gene expression [9]. They are potent modulators of diverse biological processes and pathologies comprising 1% to 5% of mammalian genes, including those liver-specific miRNAs involved in metabolic adaptation and energy homeostasis [10]. The function of miRNAs in energy metabolism was first studied in *Drosophlia melanogaster*, and revealed that miR-14 play a critical role in regulation of triacyglyceride metabolism on the whole-animal level [11]. miRNAs also safeguard insulin expression and secretion, thereby contributing to regulate adipocyte differentiation and maintenance of glucose homeostasis [12]. The liver-specific miRNA miR-122 is reported to be involved in the regulation of lipid metabolism and plays a prominent role in cholesterol and fatty acid metabolism [13]. Alterations of fatty acid metabolism-related mRNA levels for genes such as *ACACA*, *ACACB*, *ACLY*, *FASN*, *LIPC*, *SCD1* were proposed as candidate biomarker for metabolic disease in mice even in cattle for anabolic steroid abuse screening [11]. Recently the importance of miRNA-directed gene regulation are coming into focus as more miRNAs and their targets were detected in the liver of dairy cow in negative energy balance [14]. In the case of beef cows, both liver specific and ubiquitously expressed miRNAs have been also reported [15]. In response to hypoxia, hypoxia inducible factor-1 alpha (HIF-1α) has recently been reported to induce transcription of miR-210 contributing to mitochondrial respiration and, thus, modulate a fundamental shift in cellular metabolism [16]. Together, these findings suggested a strong connection between miRNAs and energy metabolism. However, whether miRNAs play a role in the energy deficiency of yak at high altitude remains to be elucidated.

To address this question, we employed next-generation sequencing to characterize the potential roles of miRNAs in liver of yak under nutrition stress. The regulatory networks underlying interactions of miRNAs with their targeted genes and biological pathways were inferred to reveal their regulatory functions in energy metabolism.

## MATERIALS AND METHODS

### Ethics statement

All samples were collected in strict accordance with the code of ethics approved by the Animal Welfare Committee of the College of Animal Science and Technology of Gansu Agricultural University (GSAU-AEW-2023-0215).

### Animals and samples collection

The experiments were conducted in cold grazing season on the alpine meadows of eastern part of the Qinghai-Tibet Plateau. The vegetation at the alpine sites is typical of species-rich alpine meadows, and is dominated by species such as by clonal *Kobresia sp*.*Festuca ovina*, *Poa poophagorum*, *Roegneria nutans*, *Agrostis sp*., *Saussurea sp*. And *Anemone rivularis*. This area is 3,200 to 4,800 m above sea level, with a dry, cold winter climate. Typically, transhumance farming defined by switching between different seasonal pasture sites is practiced, with spring and winter pastures belonging to one type, but being divided by fences as different seasonal pastures. A total of three 3-year-old domesticated yaks with body weight of 165±10 kg were chosen randomly from the same herds without any feed supplement. The yak tissue samples of liver were rapidly taken from the selected animals after slaughter and wrapped in a freezing tube, frozen in liquid nitrogen, and stored at −80°C until RNA isolation.

### Small RNA library construction and sequencing

Total RNA enriched for small RNA was extracted from 1 mg of frozen tissue from each of the three samples using the Trizol reagent (Invitrogen, Carlsbad, CA, USA) according to the manufacturer’s protocol. The RNA extract was stored at −80°C. RNA integrity was measured using the 2100 Bioanalyzer (Agilent Technologies, Santa Clara, CA, USA) and the Agilent Small RNA Kit (Agilent Technologies) was used for miRNA quantification. Small RNA libraries were constructed at BGI (Shenzhen, China) under contract and sequenced on an Illumina HiSeq2000 platform.

### Sequencing data analysis and identification of miRNAs

After adaptor trimming, removal of low-quality sequences and orphan reads, the length distribution and counts were obtained. The reads of 18 to 32 nt in length were kept for mapped to the latest bovine genome assembly (http://hgdownload.cse.ucsc.edu/goldenPath/bosTau6/bigZips/bosTau6.fa.gz) using the program SOAP [10]. The sequences of ncRNAs such as repeats, rRNAs, tRNAs, snRNAs, and snoRNA were genated from bovine mRNA (http://hgdownload.cse.ucsc.edu/goldenPath/bosTau6/database/refGene.txt.gz) and CDS (http://hgdownload.cse.ucsc.edu/goldenPath/bosTau6/bigZips/refMrna.fa.gz), Repeat Masker (http://www.repeatmasker.org) and Sanger Rfam data (version 10.1). Then the remaining reads were identified for the conserved miRNAs by a BLAST search against the Sanger miRBase (version 19.0). To discover potential novel miRNA precursor sequences, unique sequences that have more than 10 hits to the genome or that match to known non-coding RNAs were removed. Then, the flanking sequences (150 nt upstream and downstream) of each unique sequence were extracted for secondary structure analysis with Mfold (http://www.bioinfo.rpi.edu/applications/mfold) and evaluated by Mireap (http://sourceforge.net/projects/mireap/). The resulting structures were retained as novel miRNA candidates only if they met the criteria described by He et al [17]. Specifically, the miRNA candidates that passed Mireap were deemed as highly probable if their corresponding miRNAs were also found in the small RNA libraries. After prediction, the resulting potential miRNA loci were examined carefully based on the distribution and numbers of small RNAs on the entire precursor regions. Those sequences residing in the stem region of the stem-loop structure and ranging between 20 to 22 nt with free energy hybridization lower than 220 kcal/mol were considered.

### Predicted target genes of differentially expressed miRNAs

Then we extracted 55657 conserved 3′untranslated region sequences of *Bos taurus* genes. Target genes that are potentially regulated by differentially expressed miRNAs were predicted using the consensus of two computational approaches, miRanda (http://www.microrna.org/) and RNAhybrid (http://bibiserv.techfak.uni-bielefeld.de/rnahybrid/) and TargetScan (www.targetscan.org). Given the high false positive rates for miRNA target prediction, we identified only those potential target genes that were predicted by all the three methods. More specifically, in principle of miRNA target with mRNA, we downloaded 79519 Bos taurus mRNA sequences from NCBI Refseq database. Subsequently, we first established a broad pool of potential targets by applying miRanda and RNAhybrid with a free energy of hybridization less than −20 kcal/mol. To narrow down this pool of potential targets, we used TargetScan to independently identify conserved targets with a PCT-score >0.9 and/or non-conserved targets with a context+ score>90% [18]. Target genes that were not corroborated by one of the two methods were discarded. miRanda). Furthermore, Gene Ontology (GO) annotation and Kyoto encyclopedia of genes and genomes (KEGG) pathway GO and KEGG pathway analyses were performed using DAVID bioinformatics resources (http://david.abcc.ncifcrf.gov) to identify the functional modules regulated by the miRNA. To further understand those miRNAs, the top 10 predicted miRNAs were selected for in-depth analysis of the IgA immune network.

## RESULT

### Deep sequencing of liver small RNAs

**Figure 1:**
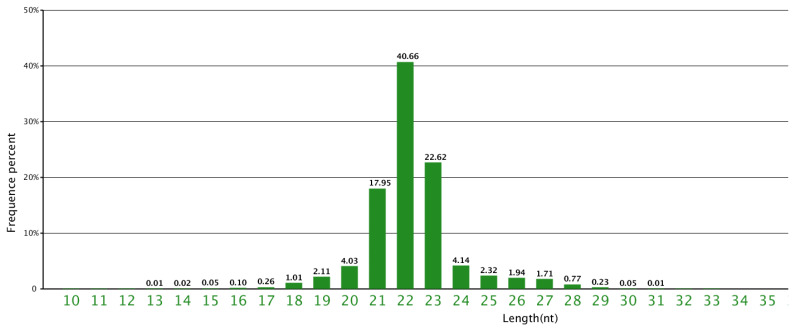
Sequence length distribution of the clean reads based on total abundance and distinct sequences.

In order to identify the miRNAs involved in the energy metabolisms of yak in the cold season, a small RNA library from a mixed pool of three domesticated yak liver samples was sequenced by the deep sequencing. A total of 17,901,522 reads were obtained. After removing the sequences shorter than 18 nt, eliminating low-quality sequences and adapter sequences, 17,562,643 clean reads were remained with 18 to 30 nt in length (Table 1), in which the majority were distributed in the range of 18 to 28 nt. The most abundant size class in the sRNA sequence distribution was 22 nt, which is consistent with the common size of miRNAs and accounted for 40.66%. This was followed by 23 nt which accounted for over 22% and 21 which accounted for nearly 18% (Figure 1).

**Figure 2:**
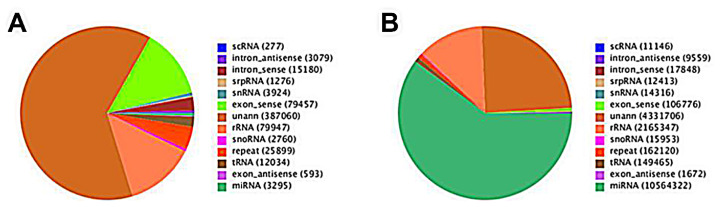
Pie charts distribution of the annotation of assigned small RNAs in liver. (A) Total small RNAs, (B) unique small RNAs.

Subsequently, in order to further analyze their expression and distribution, all of the clean reads were aligned with the bovine genome sequence using SOAP software. A total of 13,009,225 reads and 219,411 unique sRNAs were matched to the bovine genome. Then the genome-matched small RNA sequences were clustered into several RNA classes with categories as: exon_antisense, exon_sense, intron_antisense, intron_sense, miRNA, rRNA, repeat, scRNA, snRNA, snoRNA, srpRNA, tRNA, unan (unannotated) (Table 2; Figure 2). The most abundant (based on read count) RNA species in the libraries were classified as miRNAs, representing 60.15% of the library. However, after analyzing the number of unique sequences, the proportion of small RNA sequences derived from known miRNAs represented only a very small fraction of the total number of unique transcripts (0.54%). The highest fraction of unique sequences (62.96%) was unclassified small RNA sequences, which probably included novel miRNA candidates and other classes of regulatory RNAs.

### Identification of known hepatic miRNAs

To identify conserved miRNAs in our dataset, all small RNA sequences were Blastn searched against the known mature miRNAs and their precursors in the miRNA database miRBase (19.0). Currently, 662 bta-miRNA precursors and 766 mature bta-miRNAs containing 598 bta-miRNAs, 79 bta-miRNA-5p and 78 bta-miRNA-3p are deposited in miRBase. 10,564,661 total reads representing 3,367 unique sequences were annotated as miRNA candidates in the liver library, while the rest were unannotated. The miRNA candidates were then clustered into 387 categories corresponding to 278 independent genomic loci according to sequence similarity (Table 3). Further analysis identified a total of 369 known miRNAs. The largest miRNA family identified was miRNA-2284 consisting 32 members, miRNA-2285 and let-7 possessed 22 and 9 members, respectively. Many other families, such as miR-1, miR-7, miR-28, miR-31 and miR-103 had only one member.

**Figure 3:**
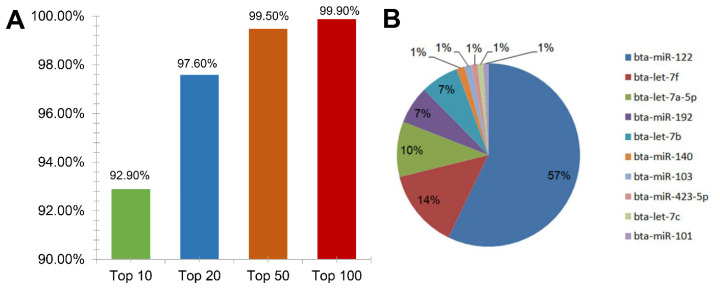
Distribution of miRNA. (A) The distribution of miRNA reads showed that the top 10, top 20, top 50 and top 100 miRNAs accounted for 92.9%, 97.6% and 99.5% and 99.0% of total reads. (B)The 10 most abundant hepatic miRNAs in the high altitude yaks. miRNA, microRNA.

The Illumina small RNA deep sequencing approach allows us to determine the relative abundance of various miRNA families by calculating the sequencing frequency. A highly expressed miRNA will likely have a large number of sequenced clones, and several miRNAs (such as bta-miR-122 and bta-let-7f) were abundant expressed more than hundreds of thousands of sequence reads. There were 28 miRNAs with more than 10,000 counts and 131 miRNAs with less than 10 counts. The top 10, top 20, top 50 and top 100 miRNAs accounted for accounted for 92.9%, 97.6% and 99.5% and 99.0% of total reads, respectively (Figure 3A). The top 10 miRNAs were bta-miR-122, bta-let-7f, bta-let-7a-5p, bta-miR-192, bta-let-7b, bta-miR-140, bta-miR-103, bta-miR-423-5p, bta-let-7c and bta-miR-101. The liver miRNA expression profile was dominated by bta-miR-122, which accounted for 57.2% of all highly expressed miRNAs. This was followed by bta-let-7f which accounted for 14% and bta-let-7a-5p which accounted for over 10% of the most abundantly expressed miRNAs. bta-miR-192 and bta-let-7b approximately to 7% and the remaining five most highly abundant miRNAs made up ~1.0% each (Figure 3B).

**Figure 4:**
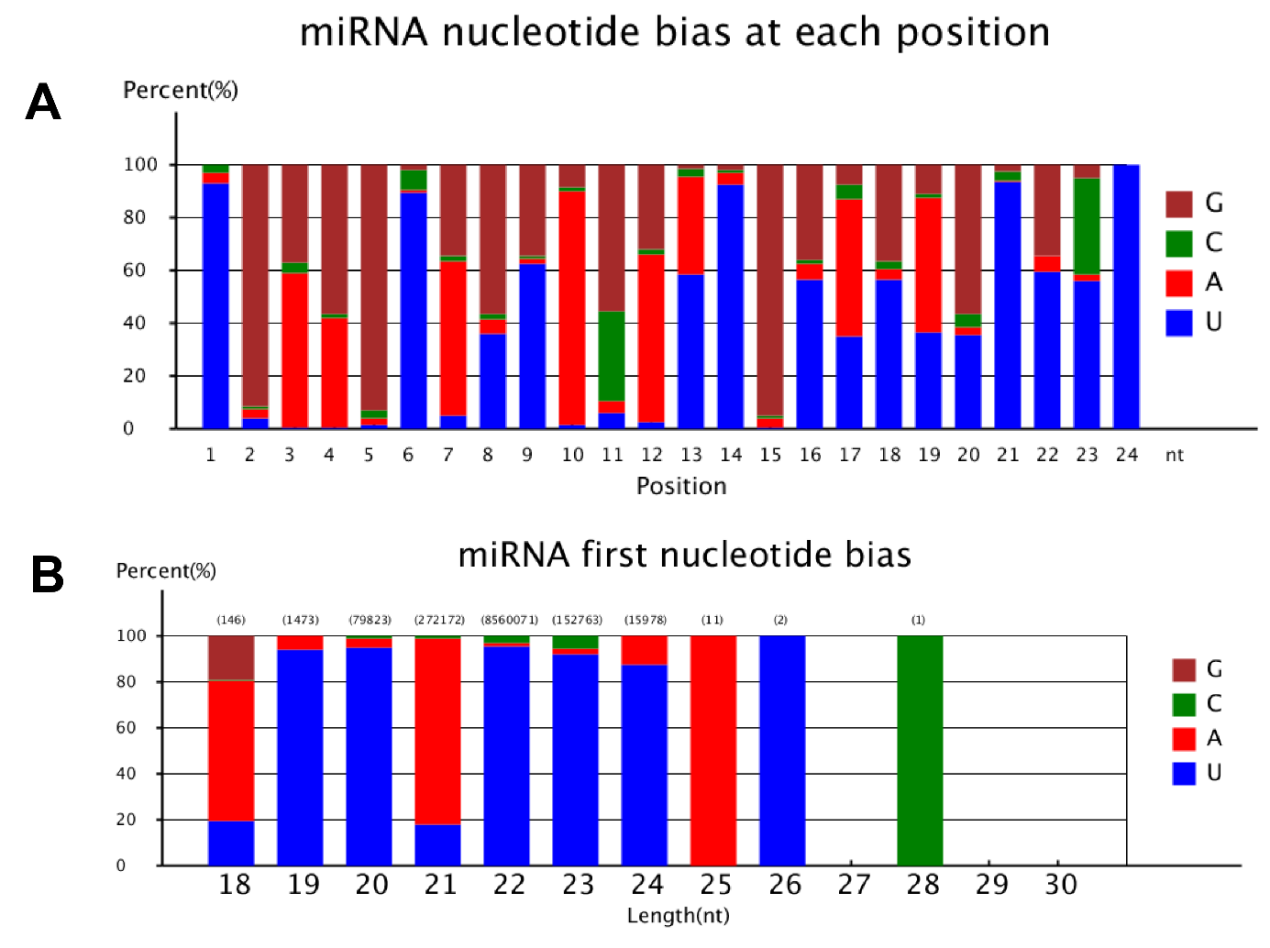
Nucleotide bias of sRNA tags. (A) MiRNA nucleotide bias at each position, (B) miRNA first nucleotide bias. miRNA, microRNA.

To better characterize and profile the identified known miRNAs, we conducted nucleotide bias analysis. The first nucleotide at the 5′ end of organ-specific miRNAs of any length was predominantly U with a frequency of 93.7%, 94.8%, 95.1% and 91.7% in 19 nt, 20 nt, 22 nt and 23 nt miRNAs. Likewise, G and A were the only preferred nucleotide at the 5′ end of 21 nt long miRNAs, although very short miRNAs that were 21 nt long were rare in yak. The base composition at each position of all mature miRNAs revealed a clear tendency for U being the most frequently observed nucleotide at specific sites (1, 6, 9, 14, and 24). The distribution of adenine (A)+uracil (U), accounting for an average of 61.7%, while nucleotides cytosine (C)+guanine (G) showed a high frequency at 2th (92.7%), 5th (96.3%), 6th (98.6%) and 15th (96.4%) position. Furthermore, from the second to eighth nucleotide positions, which belong to the seed region of miRNAs, livermiRNAs showed prominent C+G (56.3%) compared to A+U (43.7%) (Figure 4).

### Identification of potential novel miRNA candidates

The characteristic hairpin structure of a miRNA precursor can be used to predict novel miRNA. The Mireap software (http://sourceforge.net/projects/mireap/)was used to predict novel miRNAs by exploring the secondary structure, the Dicer cleavage site and the minimum free energy of the small unassigned high-quality reads. The following key conditions are used as criteria for assigning novel miRNAs: (1) The small unassigned high-quality reads must map to an intron region or an antisense exon region of a reference genome; (2) Sequence structures must satisfied the following two criteria: hairpin miRNAs can fold secondary structures and mature miRNAs are present in one arm of the hairpin precursors; (3) The mature miRNA strand and its complementary strand have 2-nucleotide 39 overhangs; (4) Hairpin precursors lack large internal loops or bulges; (5) The secondary structures of the hairpins are steady, with a free energy of hybridization lower than or equal to 218 kcal/mol. A total of 4,331,706 unannotated sequences were used to predict novel miRNAs. In total, 41 potential novel miRNA candidates with lengths ranging from 20 to 24 nt and reads ranging from 3 to 358 were obtained from liver libraries. These pre-miRNAs possessed a typical stem-loop structure and free energy ranging from −57.9 kcal/mol to −18.0 kcal/mol.

### Prediction and annotation of miRNA target genes

To comprehensively illustrate the biological processes and physiological functions of the miRNAs, we selected the top 10 miRNAs for target prediction using the integrating computational approaches of RNAhybrid (http://bibiserv.techfak.uni-bielefeld.de/rnahybrid/), miRanda (http://www.microrna.org/) and TargetScan (http://www.targetscan.org/). Given the high false positive rates for miRNA target prediction [14], we identified only those potential target genes that were predicted by any two of the three methods, and target genes that were not corroborated were discarded. In total 2286 unique potential transcripts were identified in liver for the top 10 miRNAs, respectively. To fully inspect the function of these miRNAs, GO term and KEGG pathway annotation of the predicted miRNA targets were performed using the DAVID gene annotation tool (http://david.abcc.ncifcrf.gov/). The miRNA target genes were categorized according to biological process, cellular component, and molecular function. GO term annotation results showed that cellular process, single-organism process, binding and cell were the most significantly enriched terms. Intriguingly, the metabolic process and biological regulation are also significantly enriched GO terms, suggesting that these genes may play important roles in energy homeostasis of yak under the harsh environment.

KEGG analysis identified 59 pathways that were over-represented, suggesting that these pathways are significantly regulated in the energy deficit investigated in this study. The over-represented miRNA targets belong to the MAPK signaling pathway and phosphoinositide 3-kinase (PI3K)-Akt signaling pathway, which is known to be involved in the regulation energy generating through fatty acid synthesis and glycolysis [7]. The data also highlighted pathways associated with cancer, suggesting that genes involved in cell cycle progression and cell proliferation are targeted by the differentially expressed miRNAs. In summary, the GO term and KEGG pathway annotations for the predicted miRNA targets using the DAVID gene annotation tool further illustrate the likely roles for these miRNAs during energy metabolism.

### Targets miRNAs in PI3-Akt interaction network

**Figure 5:**
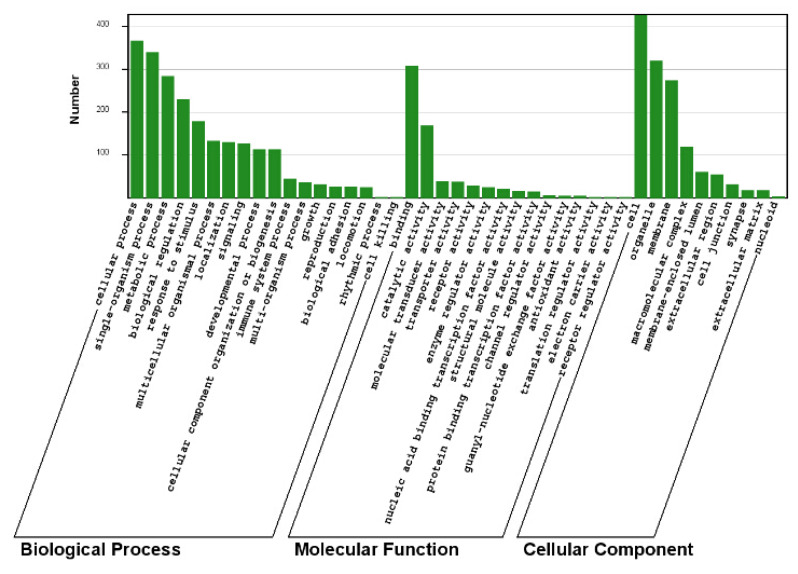
Nucleotide bias of sRNA tags.

As shown by GO and KEGG analysis, many of the identified miRNAs were predicted to participate in lipid and energy metabolism, similar to findings of other studies [11]. To further understand those miRNAs, the top 20 predicted miRNAs were selected for in-depth analysis of the PI3K-Akt interaction network. The results showed that 11 of the top 20 miRNAs are likely involved in the PI3K-Akt signaling pathway and may target about 71 related genes (Figure 5). These results suggest that liver miRNAs take part in regulation of the PI3K-Akt network by hormones and by nutrients such as glucose and fatty acids of yak. In addition, several miRNAs shared the same target gene. Interestingly, all of the let-7 family members (let-7a, let-7b, let-7c and let-7f) could target AKT2. Those miRNAs are proposed to play a key role in IgA production in the piglet digestive tract and deserve further exploration, as mucosal immunity is critically important for the protection of newborn piglets.

## DISCUSSION

Natural selection, imposed by hypoxia and cold, can have profound effects on energy generation and expenditure strategies in animals that native to high altitude. One such vertebrate species, Himalayan yak (*Bos grunniens*) separated from cattle (*Bos taurus*) approximately to 5 million years ago has successfully adapted by displaying a circannual rhythm to better adapt to the harsh environment of high altitude (~3,500 to 5,500 m) [19]. To date, hepatic miRNAs have been identified as essential mediators of energy metabolism through their function in modulation of glucose and lipid homeostasis, but a clear understanding of their role in the regulation of cellular metabolism in yak under harsh environment remains elusive. To dissect the role of hepatic miRNA in the regulation of energy deficiency in yak, the animals grazed in winter highlands selected as the model for exposed to the combined effects of nutrition stress and cold at high altitude. From using this model, we provide a comprehensive hepatic miRNA expression profiles via a deep sequencing approach. We found in total 369 known miRNAs (miRBase 19.0) and 387 pre-miRNAs producing 287 mature miRNAs. GO and KEGG pathway analyses show that those miRNAs may participate in many different metabolic-related processes. An analysis of the top 20 miRNAs showed that 11 of them may be involved in many regulatory aspects of the PI3K-Akt network. To the best of our knowledge, this is the first liver even yak miRNA-seq profiling study of moderate yak adaptation on the QTP.

In this study, objective preliminary analysis of the cDNA library has shown that 22-nt size class in liver of yak is the major type of sRNA, which is consistent with the majority of sRNA-lengths in bovine [20], and other ruminant species [21]. The mature miRNAs also showed a similar trend, which consistent with the typical size range of small RNA generated by Dicer cleavage. The total rRNA is regarded as a sign for the sample quality, and should be less than 60% in plant and nearly to 40% in animal tissues [21]. In the present study the total rRNA was 12.33%, indicating that the liver samples used were of a high quality.

The most dominant among the ten highly abundant miRNAs in our study, miR-122 is a liver-specific conserved miRNA. This dominance of expression by miR-122 is consistent with previous studies in dairy [19] and beef cows [22] where miR-122 was reported to be only expressed in liver, when compared with other tissues, and constituted more than 57% of all the miRNA reported in liver [22]. A recent study to indentify putative targets of differentially expressed miRNAs among differentially expressed hepatic genes in dairy cows in negative energy balance has revealed that tissue specific miR-122 and liver enriched miR-192 are two of the most abundant miRNAs [6]. MiR-122 reaches approximately 70% of the total miRNA population in the liver, and notably, its expression is sharply up-regulated in both mouse and human liver during embryonic development [23]. Historically, miR-122 is the first miRNA identified to regulate liver homeostasis and lipid metabolism and plays a prominent role in cholesterol accumulation and fatty acid metabolism [24]. Microarray analysis revealed reduced hepatic expression of a range of genes involved in the regulation of lipid biosynthesis such as acetyl-CoA carboxylase β (ACC2), stearoyl-CoA desaturase (SCD1), and adenosine triphosphate (ATP) citrate lyase (ACLY). Interestingly, most of the identified genes seem to be indirect targets of miR-122 because they lack seed sequences for miR-122, providing an explanation for their observed down-regulation in response to miRNA inhibition whereas the opposite would be expected for direct miRNA targets [25], suggests that miR-122 may negatively regulate a transcriptional repressor.

The next two most abundant circulating miRNA in our panel was let-7 family including let-7f and let-7a-5p. All let-7 family members are believed to exert similar functions because they share a common seed region (nucleotides 2 to 8), which mediates miRNA interaction with target mRNAs. The let-7 family members have been associated with hepatic development and disorders as well as glucose and insulin metabolism [26]. miR-192, which is also enriched in the liver in the present study and plays an important role in cellular responses to glucose stimulus, but is also expressed in kidney and gastrointestinal tract. miR-140, which is also among the highly expressed miRNAs in this study, has been reported to be abundantly expressed in human and mouse liver with implications in liver function and disorders [27]. Furthermore, miR-103 were previously predicted by bioinformatics to affect multiple mRNA targets in pathways that involve cellular acetyl-CoA and lipid levels [28], which were significantly upregulated in ob/ob mice, suggesting the potential role of in the pathogenesis of nonalcoholic fatty liver disease and confirm central importance to glucose homeostasis and insulin sensitivity, was only detected at a moderate level in yak in our study.[Fig f6-ab-24-0756]

The hepatic expression of numerous genes including those involved in glycolysis, lipid metabolism, and carbohydrate metabolism may be altered in response to acute and chronic exposure to high altitude [29]. PI3-kinases were the first mammalian lipid kinases discovered and since then multiple mammalian PI3-kinases have been identified. They are responsible for coordinating a diverse range of cell functions including cell growth, proliferation, survival, metabolism and glucose homeostasis [30]. Recent studies suggest that PI3K is also regulated by hormones and by nutrients such as glucose and fatty acids. Furthermore, activation of the P13K-Akt signaling pathways may be one mechanism by which cells adapt and survive under conditions of hypoxia [31]. In the present study, some miRNAs were predicted to target genes such as *AKT2*, *EIF4E2, JAK1*, *MAPK3*, *MTOR*, *PDPK1* involved in processes of the PI3K-Akt network, which play an important role in regulating glucose homoeostasis or lipid metabolism.

To better understand the functions of the identified miRNAs in lipid metabolism and adipogenesis, putative targets of the highly expressed top 20 miRNAs were use for PI3K-Akt network analysis. Glycogen synthase 1 (GYS1), as a predicted target gene of miR-122 regulation, which involved in cholesterol, fatty acid, and lipid metabolism [11]. Although its essential role as an energy reserve is common to all cells, there are differences in glycogen function between tissues. Liver glycogen contributes primarily to blood glucose homeostasis, being synthesized during periods of nutritional sufficiency, and subsequently converted to glucose, which is released into the bloodstream to counteract hypoglycaemia [32]. When oxygen becomes limiting, cells reduce mitochondrial respiration and increase ATP production through anaerobic fermentation of glucose. The HIFs play a key role in this metabolic shift by regulating the transcription of key enzymes of glucose metabolism. GYS1 gene induction correlated with a significant increase in glycogen synthase activity and glycogen accumulation in cells exposed to hypoxia [33]. Since fatty acid oxidation is limited and glycogen supplies glucose for anaerobic glycolysis, glucose assumes a more important role as a fuel for the heart in yaks. Significantly, knockdown of either HIF1a or GYS1 attenuated hypoxia-induced glycogen accumulation, while GYS1 overexpression was sufficient to mimic this effect, indicate that GYS1 regulation by HIF plays a central role in the hypoxic accumulation of glycogen [33].

The most of let-7 family was predict of target AKT2, primarily through glucose uptake in response to insulin through the translocation of glucose transporter type 4 (GLUT4) to the plasma membrane. Activation by AKT can also affect glucose by increasing the conversion of glucose to glucose-6-phosphate. This can either be stored through conversion back to glucose by glucogen synthase, or it can be catabolized to produce energy through glycolysis. The kinase AKT inhibits GSK3β from (in turn) inhibiting the glucogen synthase kinase activity, thereby stimulating the glucogen synthesis. However, AKT may also stimulate glycolysis through HIF-α. The lipid metabolism is also regulated by AKT through the inhibition of GSK3β which thereby promote the expression of genes involved in the cholesterol and fatty acid biosynthesis [34]. Thrombospondin1 (THBS1) was also the predicted target of let-7 family and it is noteworthy of differentially-expressed genes related to fatty acids metabolism in liver, which was previously reported as associated with the profile of intramuscular fatty acid composition in a genome-wide association study (GWAS) and RNA-Seq in pigs [35]. Therefore, they can be considered as interesting candidate genes and this suggest their role in the fatty acid metabolism processes in liver [35]. *PDK1* (3-phosphoinositide-dependent protein kinase-1), were identified as possible targets of three miRNAs of let-7 family in our study, which plays an important role in regulating glucose homoeostasis and controlling expression of insulin-regulated genes, and a deficiency of the PDK1 pathway in the liver could contribute to development of diabetes, as well as to liver failure. Let-7a, let-7c, miR-181b, miR-185, miR-378 and miR-423-5p were predicted to target the evolutionarily conserved mechanistic Target of mTOR (Rapamycin), is a member of the PI3K related kinase (PIKK) family, functions as a molecular sensor of metabolism and cellular homeostasis and integrates environmental signals by altering the cellular metabolic processes [36]. More recent work indicates that mTOR Complex 1 (mTORC1) plays a significant role in protein synthesi, lipid biosynthesis, and inhibition of triacylglycerol lipolysis [37]. Thus it may have a significant impact on maintaining metabolic homeostasis in the whole body. mTORC1 enhances the translation of HIF-1α, a transcription factor, that in turn regulates the transcription of genes encoding glycolytic enzymes and glucose transporters [38]. As a result, mTORC1 promotes glucose uptake and activation of glycolysis to generate energy. The ability to control the rates of metabolic processes in response to changes in the internal or external environment is indispensable for all living cells. Mechanisms that are essential for metabolic control and maintenance of homeostasis are complex and involve transcriptional, translational, posttranslational, and allosteric regulation. Our results indicate that some miRNAs add a new level of regulation and fine tuning for gene expression that is likely to be important for a wide range of cellular functions, including signaling and metabolic control. However, their regulatory mechanisms warrant furher study.

## CONCLUSION

In conclusion, the present study revealed 369 known and 41 novel miRNA candidates, most of which were predicted to be involved in the regulation of lipid as well as glucose homeostasis via the PI3K-Akt interaction network for the yak under deficiency energy. These findings contribute to an increased understanding of the roles of miRNAs in liver and to building the foundation for understanding their physiological functions and regulatory mechanisms for yak to better adapt to the harsh environment.

## Figures and Tables

**Figure 6 f6-ab-24-0756:**
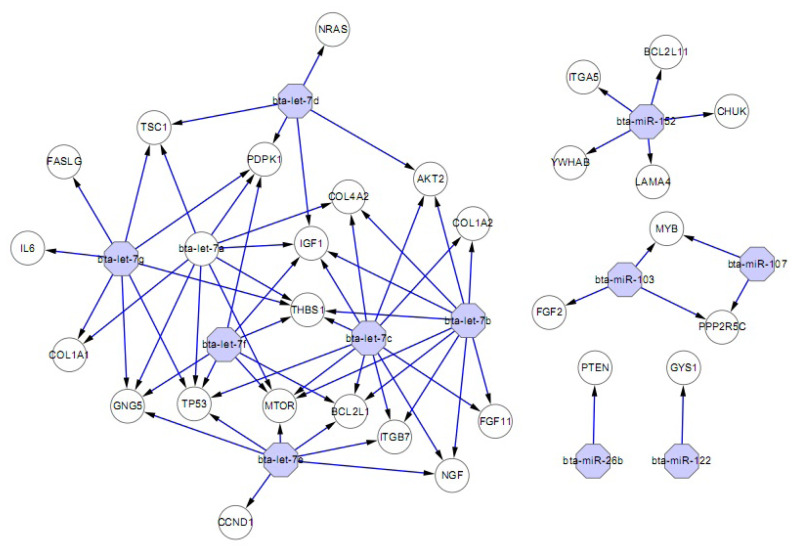
MiRNAs targeting the PI3-Akt interaction network. The top 20 miRNAs were analyzed, and 11 of them were found to participate in PI3-Akt interaction network, involving 20 target genes. Different colors and shapes represent various relationships between miRNAs and genes. miRNA, microRNA.

**Table 1:** Summary of small RNA sequencing date

Type	Liver	

	Count	Percentage (%)
Total_reads	18,000,000	
High_quality	17,901,522	100
3′Adapter_null	5,680	0.03
Insert_null	3,840	0.02
5′Adapter_contaminants	250,400	1.40
Smaller_than_18nt	78,887	0.44
PolyA	72	0.00
Clean_reads	17,562,643	98.11

**Table 2:** Distribution of the genome-mapped sequence reads in small RNA libraries

Type	Heart			

	Unique sequences		Reads	
	
Total	614781	100%	17562643	100%
Exon_antisense	593	0.10%	1672	0.01%
Exon_sense	79457	12.92%	106776	0.61%
Intron_antisense	3079	0.50%	9559	0.05%
Intron_sense	15180	2.47%	17848	0.10%
MiRNA	3295	0.54%	10564322	60.15%
rRNA	79947	13.00%	2165347	12.33%
Repeat	25899	4.21%	162120	0.92%
scRNA	277	0.05%	11146	0.06%
snRNA	3924	0.64%	14316	0.08%
snoRNA	2760	0.45%	15953	0.09%
srpRNA	1276	0.21%	12413	0.07%
tRNA	12034	1.96%	149465	0.85%
Unann	387060	62.96%	4331706	24.66%

**Table 3:** Summary of known miRNA in each sample

	miR	miR-5p	miR-3p	Pre-miRs	Unique matched to pre-miRs	Read matched to pre-miRs
Known miRs	598	79	78	766		
LIver	278	42	48	387	3367	10564661

miRNA, microRNA.
